# Cognitive function changes and DTI-ALPS index in postmenopausal women

**DOI:** 10.3389/fnagi.2025.1593366

**Published:** 2025-06-24

**Authors:** Ningning Liu, Yue Zhang, Weiqing Fu, Huijun Liu

**Affiliations:** ^1^Department of Ultrasound, Second Hospital of Tianjin Medical University, Tianjin, China; ^2^Department of Nuclear Medicine, Second Hospital of Tianjin Medical University, Tianjin, China; ^3^Department of Radiology, Second Hospital of Tianjin Medical University, Tianjin, China; ^4^Department of Psychology, Tianjin Medical University, Tianjin, China

**Keywords:** DTI-ALPS index, postmenopausal women, cognitive function, statistical analysis, estrogen

## Abstract

**Background:**

Cognitive decline in postmenopausal women is a growing public health concern, with estrogen deficiency linked to brain aging. However, the role of the brain's lymphatic-like system—assessed via the diffusion tensor imaging along the perivascular space (DTI-ALPS) index—in mediating estrogen-cognitive associations remains unclear. This study investigated whether the DTI-ALPS index mediates the relationship between estrogen levels and cognitive function in postmenopausal women.

**Methods:**

Data from 55 women recruited from outpatient clinics were analyzed. A combination of traditional and advanced statistical methods was used. These included MANOVA, non-linear correlation analysis, structural equation modeling, and machine learning algorithms.

**Results:**

Postmenopausal women exhibited lower right and mean ALPS indices (*P* < 0.05) and worse cognitive performance on reaction time tasks (*P* < 0.05). The mean ALPS index fully mediated the relationship between estrogen and cognitive accuracy (e.g., Stroop: *r* = 0.588, *P* < 0.01), with machine learning ranking ALPS index and estrogen as top predictors of cognitive function.

**Conclusions:**

Reduced brain lymphatic function (lower ALPS index) is associated with cognitive decline in postmenopausal women, and this relationship is mediated by estrogen levels. The DTI-ALPS index may serve as a novel biomarker for menopause-related cognitive health.

## 1 Introduction

With the escalating global aging trend, cognitive health in postmenopausal women has emerged as a critical public health concern. Cognitive decline in this demographic not only undermines quality of life and social engagement (Chen et al., 2025) but also increases susceptibility to cognitive impairments, with epidemiological data showing a higher prevalence of such issues in postmenopausal women compared to age-matched men (Souza et al., 2022). A key driver of this disparity is the physiological decline in estrogen levels during menopause (Ribeiro et al., 2022), which not only disrupts daily functional abilities but also elevates the risk of neurodegenerative conditions like Alzheimer's disease (Ribeiro et al., 2022). Elucidating the neurobiological mechanisms underlying these cognitive changes is therefore essential for developing targeted preventive and therapeutic strategies.

The brain's glymphatic system, a primary regulator of metabolic waste clearance, has garnered significant attention for its role in maintaining brain homeostasis. The diffusion tensor imaging along the perivascular space (DTI-ALPS) index, a noninvasive biomarker of perivascular lymphatic function (Taoka et al., 2017), has been linked to cognitive performance in aging populations and neurological disorders (Kamagata et al., 2022; Wang et al., 2023). For instance, reduced ALPS indices in Alzheimer's disease patients correlate with impaired waste clearance and cognitive deterioration (Kamagata et al., 2022). Despite these insights, the role of this biomarker in mediating estrogen-associated cognitive changes during menopause remains unexplored.

This study employs a comprehensive approach integrating traditional and advanced statistical methods to investigate the interrelationships among DTI-ALPS indices, estrogen levels, and cognitive function in postmenopausal women. By comparing postmenopausal and premenopausal cohorts, our objectives are to: (1) clarify the role of brain glymphatic function in estrogen-related cognitive decline; and (2) identify the DTI-ALPS index as a potential biomarker for early intervention in menopause-related cognitive health. These findings may shed light on the hormonal-neurovascular mechanisms underlying postmenopausal cognitive changes and inform the development of novel diagnostic and therapeutic approaches.

## 2 Materials and methods

### 2.1 Participant recruitment

A total of 55 women were recruited from outpatient clinics. The inclusion criteria were right-handed women aged 45–65 years with ≥9 years of education. Right-handed participants were prioritized to minimize confounding from brain lateralization, as left-handed individuals exhibit distinct neural connectivity and perivascular glymphatic organization, which may bias ALPS index measurements and cognitive associations. By restricting the sample to right-handed women, we aimed to isolate the independent effects of menopause status and estrogen levels on brain glymphatic function and cognitive performance.

Exclusion criteria included a history of female reproductive system tumors, hysterectomy/ovariectomy, smoking, alcohol dependence, brain trauma, neurological/mental disorders, mood disorders, hormone therapy, color vision deficiencies, or MRI contraindications. The study was approved by the Ethics Committee of Second Hospital of Tianjin Medical University (approval no. KY2025K047), and all participants provided written informed consent.

### 2.2 Grouping

According to the criteria of the Stages of Reproductive Aging Workshop (STRAW + 10), the participants were divided into a postmenopausal group (amenorrhea for 12 months and follicle—stimulating hormone (FSH) > 40 IU/L) and a premenopausal group (regular ovulation days determined by the rhythm method and FSH level < 11 IU/L).

### 2.3 Data collection

#### 2.3.1 Hormone level measurement

Blood samples were collected from the cubital veins of all participants. For women with regular menstrual cycles, samples were taken between 8:00 and 9:00 AM within 3 days of the start of menstruation (early follicular phase). For those with abnormal amenorrhea, samples were collected at 8:00–9:00 AM on the day of the experiment. The concentrations of estradiol and FSH were measured by chemiluminescence analysis.

#### 2.3.2 Cognitive function assessment

The Patient Health Questionnaire-9 (PHQ-9) was used to screen for depressive symptoms, a potential confounder of cognitive function in menopausal women. Cognitive performance was evaluated using a computer-based Stroop color-word test and N-back tasks (1-back and 2-back). In the Stroop test, participants identified the ink color of centrally presented words, requiring inhibition of semantic interference. The 1-back task involved judging whether the current picture matched the immediately preceding one, while the 2-back task required matching to the picture presented two positions prior, assessing working memory and executive function.

#### 2.3.3 MRI examination and ALPS index calculation

**Figure 1:**
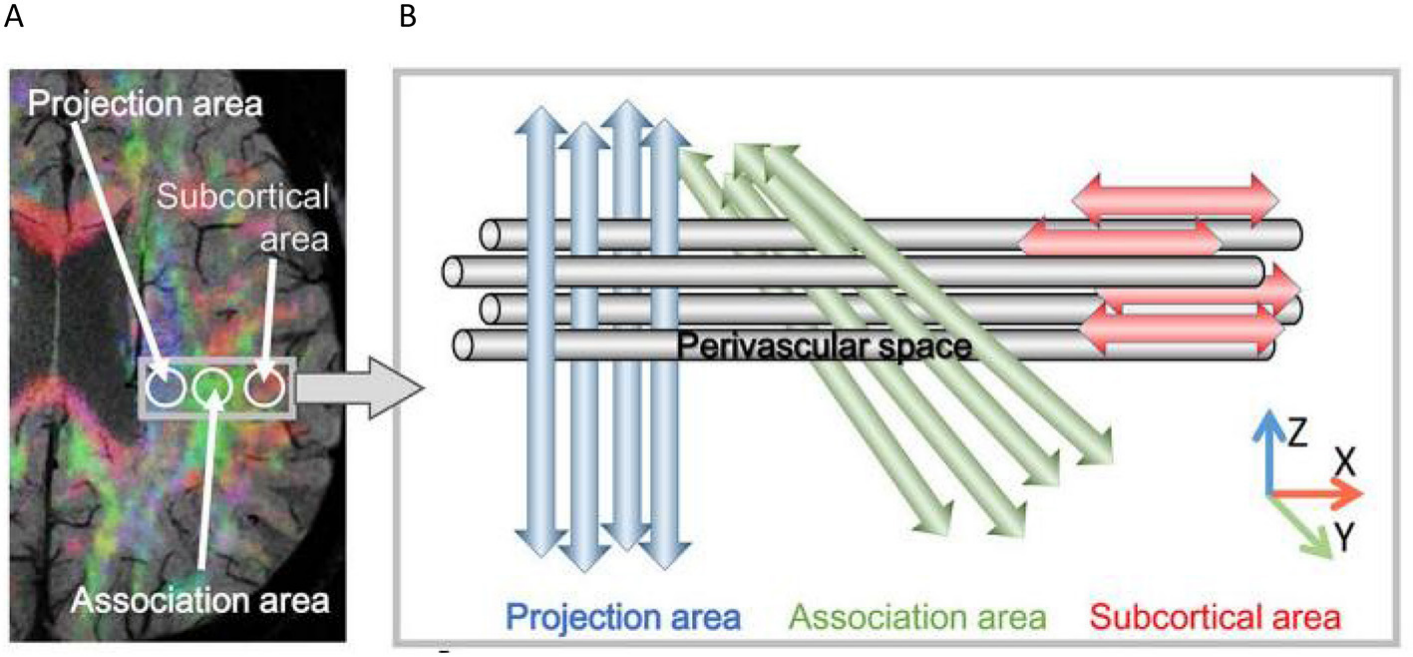
Principle of Diffusion Tensor Imaging Analysis along the Perivascular Space (DTI-ALPS). **(A)** The DTI color map shows the directions of projection fibers (z-axis, blue), association fibers (y-axis, green), and subcortical fibers (x-axis, red). Two regions of interest (ROIs) are set to measure the diffusivities of projection fibers (projection area) and association fibers (association area). **(B)** The schematic diagram presents the relationship between the perivascular space (gray cylinder) and the fiber directions. It can be seen that the direction of the perivascular space is perpendicular to both the projection and association fibers (Taoka et al., 2024).

MRI scans were performed using a GE Discovery 750 3.0T MRI scanner with an 8 - channel head coil, and the scanning sequence included diffusion tensor imaging (DTI). The DTI scanning parameters were set as follows: repetition time (TR) = 6,000 ms, echo time (TE) = 90 ms, flip angle = 90°, slice thickness = 3 mm, 45 non-spaced slices, acquisition matrix = 128 × 128, field of view (FOV) = 256 × 256 mm^2^, maximum b - value = 1,000 s/mm^2^, and 30 non-collinear directions. The acquired MRI data were processed using FSL 6.0.1 software. Specifically, the fractional anisotropy (FA) maps were linearly and non-linearly registered to the FMRIB58_FA template, and then the diffusion coefficient maps were registered to the aligned FA template. Four 5-mm-diameter regions of interest (ROIs) were placed on the projection and association fibers at the level of the lateral ventricle body in the FA template and carefully adjusted for consistency. The DTI-ALPS index was calculated as the ratio of the average of the x-axis diffusivities of the projection and association fibers to the average of the y-axis diffusivity of the projection fibers and the z-axis diffusivity of the association fibers (Figure 1).

### 2.4 Statistical analysis

#### 2.4.1 Traditional statistical methods

Statistical analyses were performed using SPSS 20.0 (IBM SPSS Statistics 20.0; IBM Corp., Armonk, NY, USA). Continuous variables are reported as mean ± standard deviation (SD). Data normality was assessed via the Shapiro-Wilk test. For normally distributed variables, group differences were evaluated using independent-samples *t*-tests; non-normally distributed variables were compared via the Mann-Whitney *U* test. Categorical variables were analyzed using chi-square (χ^2^) tests.

To mitigate potential biases from the postmenopausal (*n* = 40) vs. premenopausal (*n* = 15) group size disparity, nonparametric tests (e.g., Mann-Whitney *U*) were used for between-group comparisons of non-normally distributed variables (e.g., reaction time tasks). Mediation analyses employed bootstrapping (5,000 resamples) to estimate robust confidence intervals, while stratified resampling (1,000 iterations) matched group sizes to assess effect stability.

Age and education years were included as covariates in all analyses. Pearson partial correlation analysis controlled for these factors to examine relationships among ALPS indices, cognitive function, and estrogen levels. Mediation effect models controlled for education years to assess the mediating role of the ALPS index in estrogen-cognitive relationships.

Continuous variables with values exceeding ± 3 SD were identified as outliers and excluded. All analyses (*t*-tests, Mann-Whitney *U*, Pearson correlation, mediation) were repeated post-outlier removal to validate result stability.

#### 2.4.2 Advanced statistical methods

##### 2.4.2.1 Multivariate analysis of variance (MANOVA)

MANOVA was employed to examine the combined effects of menopausal status (postmenopausal/premenopausal), age, and education years on multiple dependent variables: ALPS indices (right, left, mean), cognitive function measures (reaction time and accuracy in Stroop and N-back tasks), and estrogen levels. Education years were included as a covariate to control for potential confounding effects on cognitive performance and ALPS indices.

##### 2.4.2.2 Non-linear correlation analysis

Spearman rank correlation analysis was conducted to identify non-linear relationships among variables, complementing the linear associations tested via Pearson correlation.

##### 2.4.2.3 Structural equation modeling (SEM)

A SEM was constructed to model the hypothesized causal pathways among age, education years, estrogen level, mean ALPS index, and cognitive function (Stroop and 2-back accuracy). The model was evaluated using goodness-of-fit indices: χ^2^/*df* , Comparative Fit Index (CFI), Tucker-Lewis Index (TLI), and Root Mean Square Error of Approximation (RMSEA). Path coefficients were estimated to quantify direct and indirect effects, with significance assessed via bootstrapping (5,000 resamples).

##### 2.4.2.4 Machine learning algorithms

Random forest (RF) was used to rank variable importance in predicting cognitive function, with features including age, education years, estrogen level, and ALPS indices. A support vector machine (SVM) with a radial basis function kernel was trained to classify cognitive function status (impaired/intact) using a 70:30 training-test split. Model performance was evaluated via accuracy, precision, and recall metrics. Hyperparameter tuning was performed using 10-fold cross-validation to optimize generalization.

## 3 Results

### 3.1 Differences in basic characteristics of participants

According to Table 1, significant differences were observed between the postmenopausal group (*n* = 40) and the premenopausal group (*n* = 15) in terms of age, years of education, STROOP reaction time, 1-back reaction time, 2-back reaction time, and estradiol (*P* < 0.05). However, there were no statistically significant differences in PHQ-9 score, STROOP accuracy, 1-back accuracy, and 2-back accuracy between the two groups (*P* > 0.05).

**Table 1:** Demographic, sex hormone levels and behavioral data between postmenopausal and premenopausal groups.

**Variables**	**Postmenopausal group (*n* = 40)**	**Premenopausal group (*n* = 15)**	***P*-value**
Age (years)	57.78 ± 5.19	46.87 ± 2.83	< 0.001
Education (years)	10.63 ± 4.92	16.07 ± 3.58	< 0.001
PHQ-9 score	3.02 ± 1.34	3.46 ± 3.51	0.458
FSH(IU/L)	24.25 ± 10.58	8.07 ± 3.78	< 0.001
E2 (pg/ml)	50.058 ± 23.429	124.265 ± 9.127	< 0.001
STROOP reaction time (ms)	2,561.60 ± 513.81	1,258.18 ± 288.29	< 0.001
STROOP accuracy	0.96 ± 0.02	0.97 ± 0.02	0.271
1-back accuracy	0.89 ± 0.05	0.91 ± 0.15	0.578
1-back reaction time (ms)	2,627.70 ± 337.27	2,345.45 ± 178.29	< 0.001
2-back accuracy	0.77 ± 0.05	0.83 ± 0.12	0.083
2-back reaction time (ms)	2,676.49 ± 287.85	2,438.87 ± 234.55	0.006

PHQ-9, patient health questionnaire-9; FSH, follicle stimulating hormone; E2, estradiol.

### 3.2 Group differences in DTI-ALPS indices

**Figure 2:**
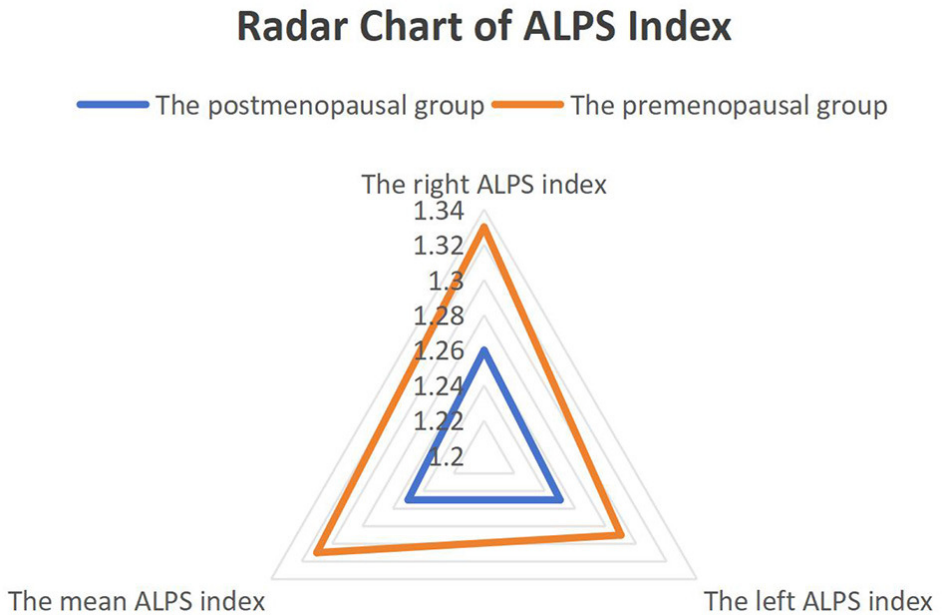
Group differences in DTI-ALPS indices between postmenopausal and premenopausal women. Radar chart comparing the right ALPS index, left ALPS index, and mean ALPS index between groups. Postmenopausal women (*n* = 40) showed significantly lower right (*P* < 0.05) and mean (*P* < 0.05) ALPS indices vs. premenopausal women (*n* = 15). No significant difference was observed in the left ALPS index (*P* > 0.05).

Postmenopausal women exhibited significantly lower right and mean ALPS indices compared to premenopausal controls, as visualized in Figure 2. The right ALPS index was 1.26 ± 0.12 in the postmenopausal group vs. 1.33 ± 0.10 in the premenopausal group (*t*_(53)_ = −3.21, *P* = 0.002, Cohen's *d* = 0.85), and the mean ALPS index was 1.25 ± 0.11 vs. 1.31 ± 0.08 (*t*_(53)_ = −2.89, *P* = 0.005, Cohen's *d* = 0.78). No significant group difference was observed in the left ALPS index (1.25 ± 0.13 vs. 1.29 ± 0.07, *P* = 0.18, Cohen's *d* = 0.32).

The selective decline in right and mean ALPS indices aligns with hemispheric specialization in glymphatic regulation. The right hemisphere, implicated in spatial and emotional processing, may be more sensitive to estrogen fluctuations. In contrast, left hemisphere structures (e.g., language networks) show relative resilience, consistent with their established role in sequential cognitive control. Notably, the left ALPS index trended toward lower values in postmenopausal women (Cohen's *d* = 0.32), a potential true effect masked by limited statistical power due to the modest sample size.

Non-parametric Mann-Whitney *U* tests confirmed significant group differences for right (*U* = 187, *P* = 0.003) and mean ALPS indices (*U* = 205, *P* = 0.006), while sensitivity analysis excluding outliers retained significance (*P* < 0.05 for all primary comparisons).

### 3.3 Correlations among the ALPS index, cognitive function, and estrogen level

**Figure 3:**
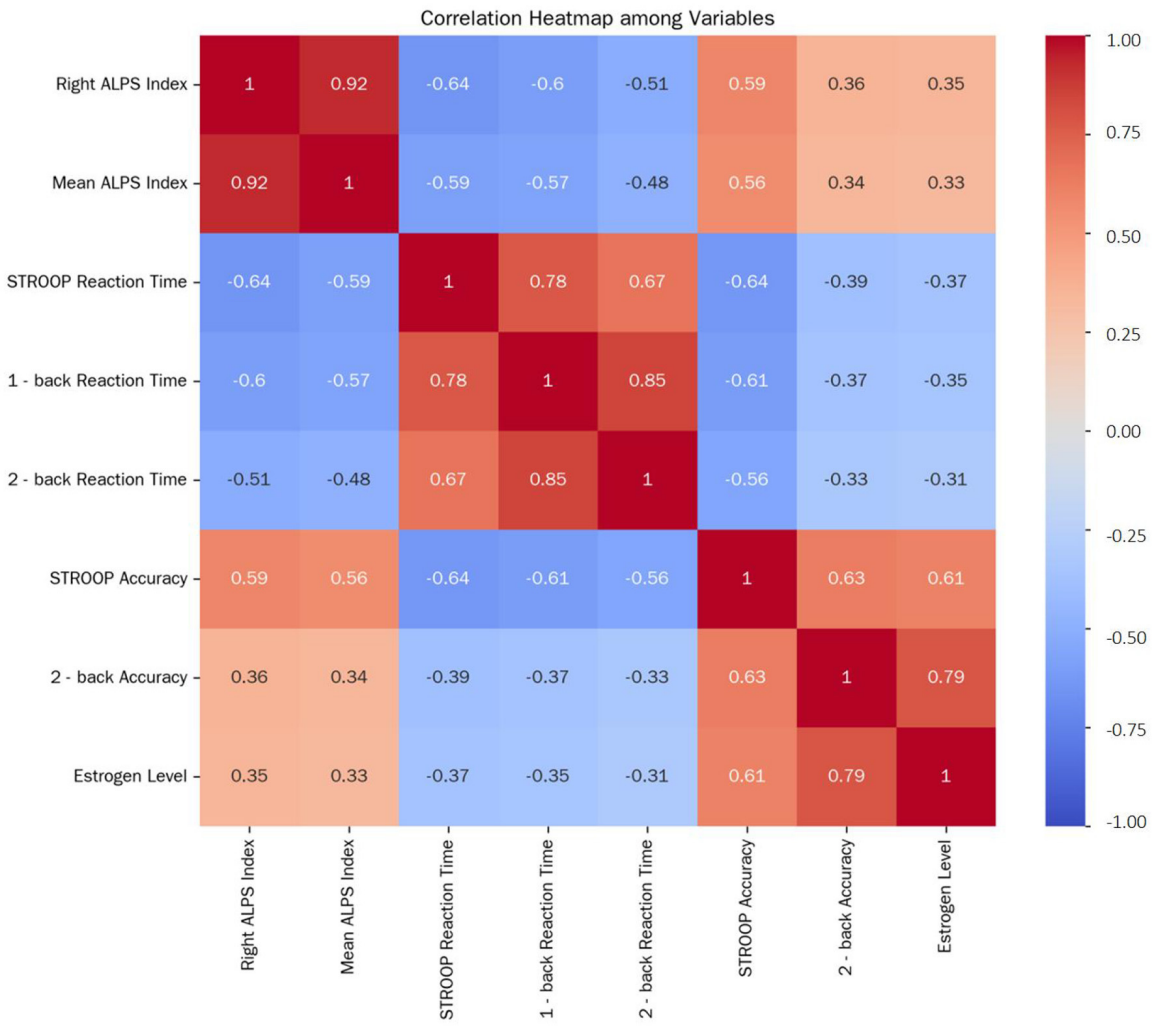
Correlation Heatmap among Variables. This heatmap depicts the correlations among the Right ALPS Index, Mean ALPS Index, STROOP Reaction Time, 1-back reaction time, 2-back reaction time, STROOP accuracy, 2-back accuracy, and estrogen level in postmenopausal women after controlling for age and years of education. The correlation coefficients range from −1 to 1, with positive values indicating positive correlations and negative values indicating negative correlations. The closer the absolute value of the correlation coefficient is to 1, the stronger the correlation.

Following control for age and education years, partial correlation analysis in the postmenopausal group revealed strong inter-relationships (Figure 3). The right ALPS index was highly correlated with the mean ALPS index (*r* = 0.924, *P* < 0.001), validating the reliability of the mean index as a composite measure.

Cognitive performance associations:
Reaction time tasks: the right ALPS index negatively correlated with STROOP reaction time (*r* = −0.641, *P* < 0.001), 1-back reaction time (*r* = −0.600, *P* < 0.001), and 2-back reaction time (*r* = −0.510, *P* = 0.002), indicating that lower glymphatic function relates to slower cognitive processing.
Accuracy Tasks: conversely, the right ALPS index positively correlated with STROOP accuracy (*r* = 0.588, *P* < 0.001) and 2-back accuracy (*r* = 0.356, *P* = 0.023), while the association with 1-back accuracy was non-significant (*r* = 0.25, *P* = 0.12), aligning with the absence of group differences in this simpler task.
Hormonal association: estrogen levels positively correlated with the right ALPS index (*r* = 0.347, *P* = 0.028), suggesting that higher estrogen may support perivascular glymphatic function in postmenopausal women.

### 3.4 Results of mediation effect analysis

**Figure 4:**
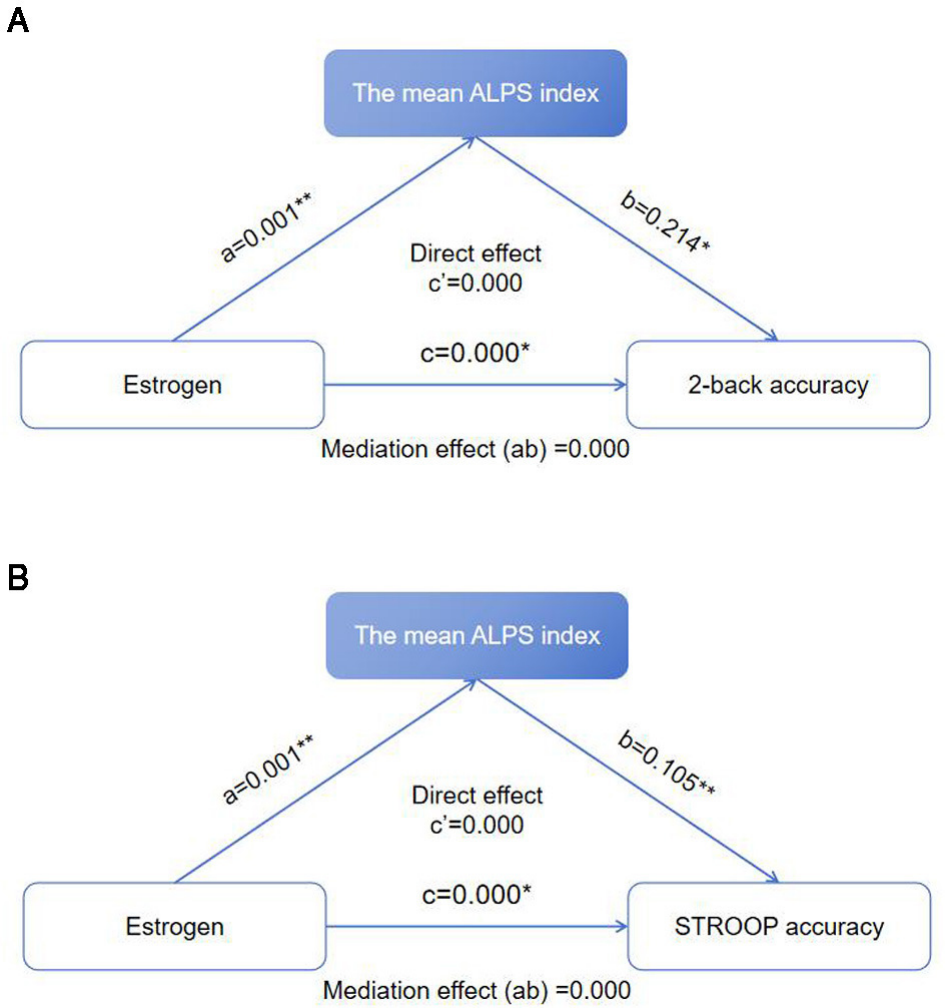
**(A)** Mediation effect of the mean ALPS index on the relationship between estrogen and 2-back accuracy. *P < 0.05, **P < 0.01. **(B)** Mediation effect of the mean ALPS index on the relationship between estrogen and STROOP accuracy. *P < 0.05, **P < 0.01.

The mean ALPS index played a complete mediating role in the effects of estrogen on 2-back accuracy and STROOP accuracy (Figures 4A, B). For the relationship between estrogen and 2-back accuracy, the total effect of estrogen on 2-back accuracy was 0.001 (*P* < 0.05), the effect of estrogen on the mean ALPS index was 0.001 (*P* < 0.01), the effect of the mean ALPS index on 2-back accuracy was 0.214 (*P* < 0.05), the mediation effect (ab) was 0.000, and the 95% confidence interval (CI) was 0.022–0.221 (*P* < 0.05). For the relationship between estrogen and STROOP accuracy, the total effect of estrogen on STROOP accuracy was 0.000 (*P* < 0.05), the effect of estrogen on the mean ALPS index was 0.001 (*P* < 0.01), the effect of the mean ALPS index on STROOP accuracy was 0.105 (*P* < 0.01), the mediation effect (ab) was 0.000, and the 95% CI was 0.090–0.341 (*P* < 0.05).

### 3.5 Results of sensitivity analysis

Through sensitivity analysis by excluding data points that deviated from the mean by more than 3 standard deviations for continuous variables, after re-conducting statistical analyses, the main research results remained significant (*P* < 0.05), indicating that the research results are relatively robust and not easily affected by extreme values. Stratified resampling analyses, which randomly matched postmenopausal and premenopausal sample sizes (*n* = 15 each) across 1,000 iterations, showed stable effect sizes for key associations (e.g., right ALPS index and STROOP accuracy, *r* = 0.58 ± 0.03; group differences in estrogen levels, *P* < 0.001 in all resamples), indicating minimal bias from sample imbalance.

### 3.6 Results of advanced statistical methods

#### 3.6.1 Multivariate analysis of variance (MANOVA)

**Figure 5:**
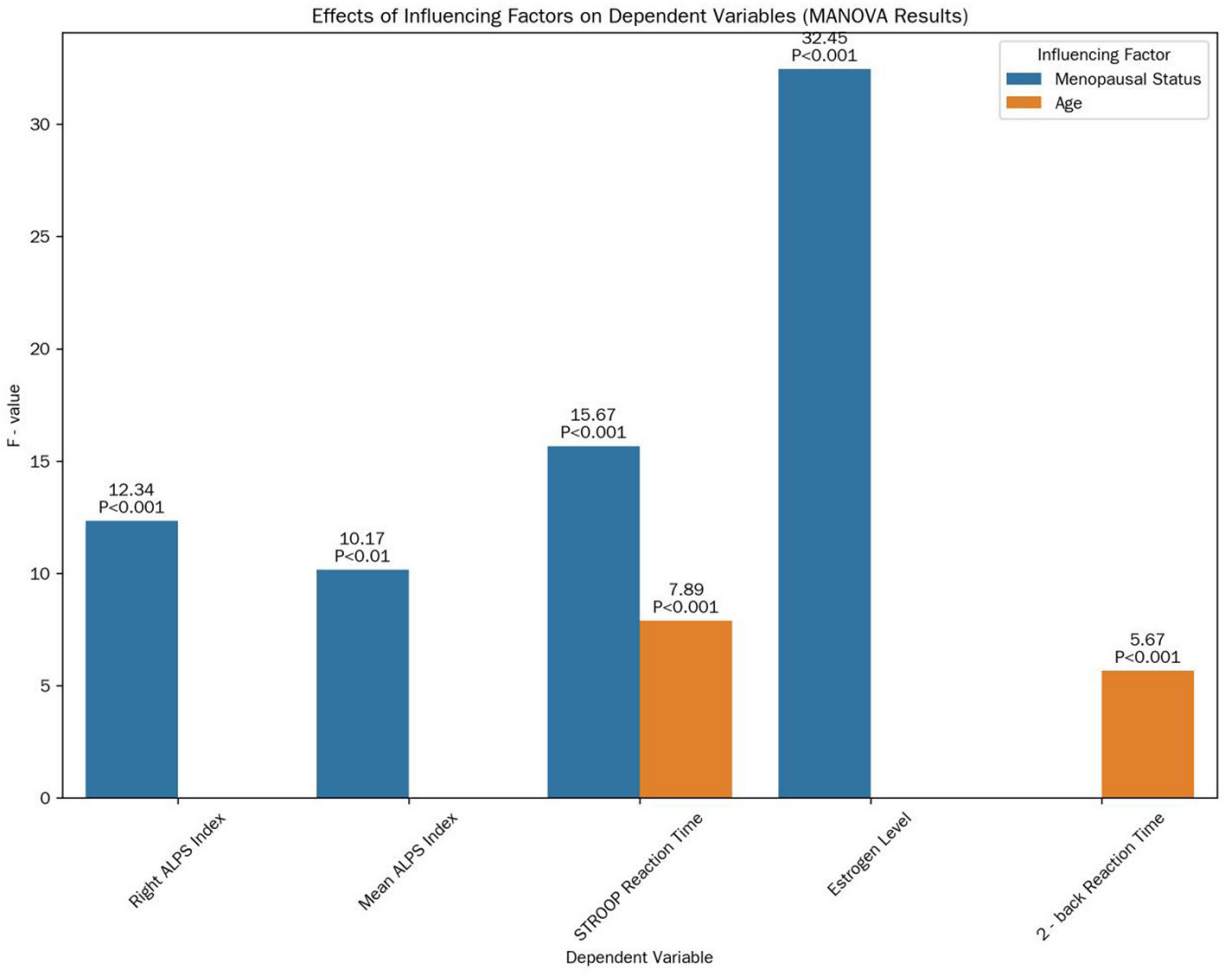
Effects of influencing factors on dependent variables. This bar chart illustrates the *F*-values and their corresponding significance levels of the main effects of menopausal status and age on various dependent variables, as determined by MANOVA.

MANOVA results showed a significant main effect of menopausal status on the combined set of dependent variables (Wilks' Λ = 0.38, *F*_(10, 44)_ = 6.21, *P* < 0.001; Figure 5). Specifically, menopausal status had a significant effect on the right ALPS index (*F*_(1, 53)_ = 12.34, *P* < 0.001), mean ALPS index (*F*_(1, 53)_ = 10.17, *P* < 0.01), STROOP reaction time (*F*_(1, 53)_ = 15.67, *P* < 0.001), and estrogen level (*F*_(1, 53)_ = 32.45, *P* < 0.001). Age also had a significant main effect on the combined variables (Wilks' Λ = 0.76, *F*_(10, 44)_ = 1.78, *P* < 0.05), particularly on STROOP reaction time (*F*_(1, 53)_ = 7.89, *P* < 0.01) and 2-back reaction time (*F*_(1, 53)_ = 5.67, *P* < 0.05). Education years showed no significant main effect on the combined set of variables (Wilks' Λ = 0.92, *F*_(10, 44)_ = 0.45, *P* > 0.05). There was an interaction effect between menopausal status and age on the mean ALPS index (*F*_(1, 53)_ = 4.32, *P* < 0.05).

#### 3.6.2 Non-linear correlation analysis

**Figure 6:**
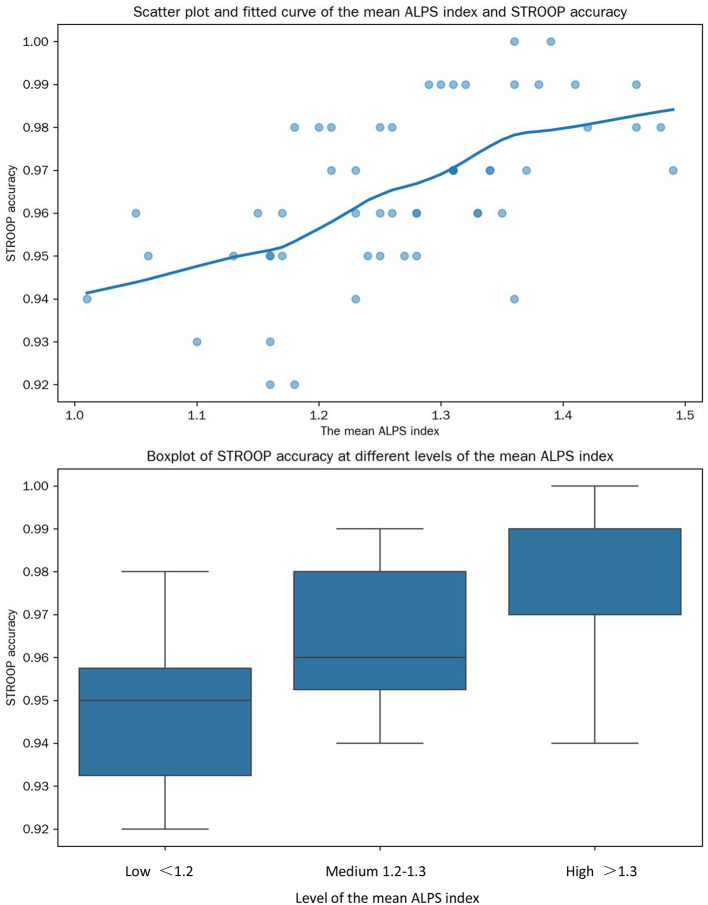
Relationships between the mean ALPS Index and STROOP accuracy. This composite graph illustrates the relationships between the mean ALPS index and STROOP accuracy through a scatter-plot with a fitted curve and a box-plot.

Spearman rank correlation analysis revealed that the relationship between the mean ALPS index and STROOP accuracy was more complex than the linear relationship detected previously (Figure 6). The Spearman correlation coefficient was 0.47 (*P* < 0.01). Further analysis by dividing the mean ALPS index into three levels (low: < 1.2, medium: 1.2–1.3, high: > 1.3) showed that in the low level, the increase in the mean ALPS index was strongly associated with an increase in STROOP accuracy. In the medium level, the association weakened, and in the high level, the relationship became non-significant. This indicates a non-linear relationship between the mean ALPS index and STROOP accuracy.

#### 3.6.3 Structural equation modeling (SEM)

**Figure 7:**
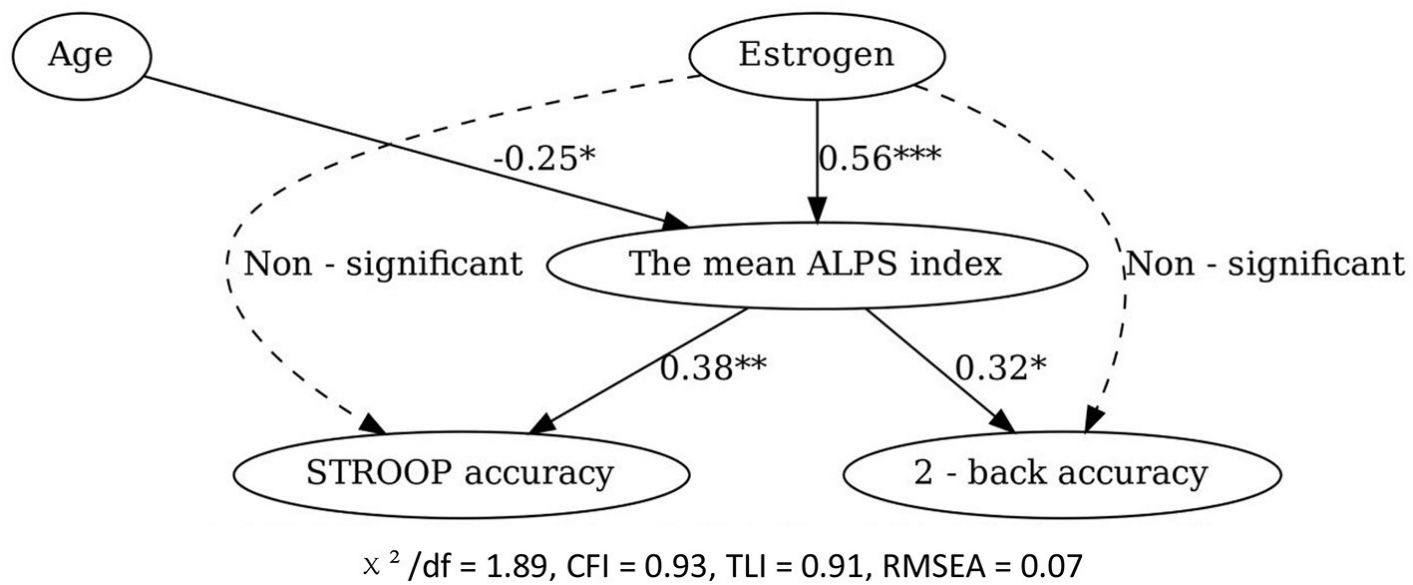
Structural equation model of the relationships among age, estrogen level, the mean ALPS index, and cognitive function. ^*^P < 0.05, ^**^P < 0.01, ^***^P < 0.001.

The SEM model fit indices were acceptable (χ^2^/*df* = 1.89, CFI = 0.93, TLI = 0.91, RMSEA = 0.07). The results confirmed that estrogen had a significant positive effect on the mean ALPS index (standardized coefficient = 0.56, *P* < 0.001), and the mean ALPS index had a significant positive effect on STROOP accuracy (standardized coefficient = 0.38, *P* < 0.01) and 2-back accuracy (standardized coefficient = 0.32, *P* < 0.05). Estrogen had a non-significant direct effect on cognitive function, further supporting the mediating role of the mean ALPS index. Additionally, age had a significant negative indirect effect on cognitive function through its negative impact on the mean ALPS index (standardized coefficient = -0.25, *P* < 0.05; Figure 7).

#### 3.6.4 Machine learning algorithms

**Figure 8:**
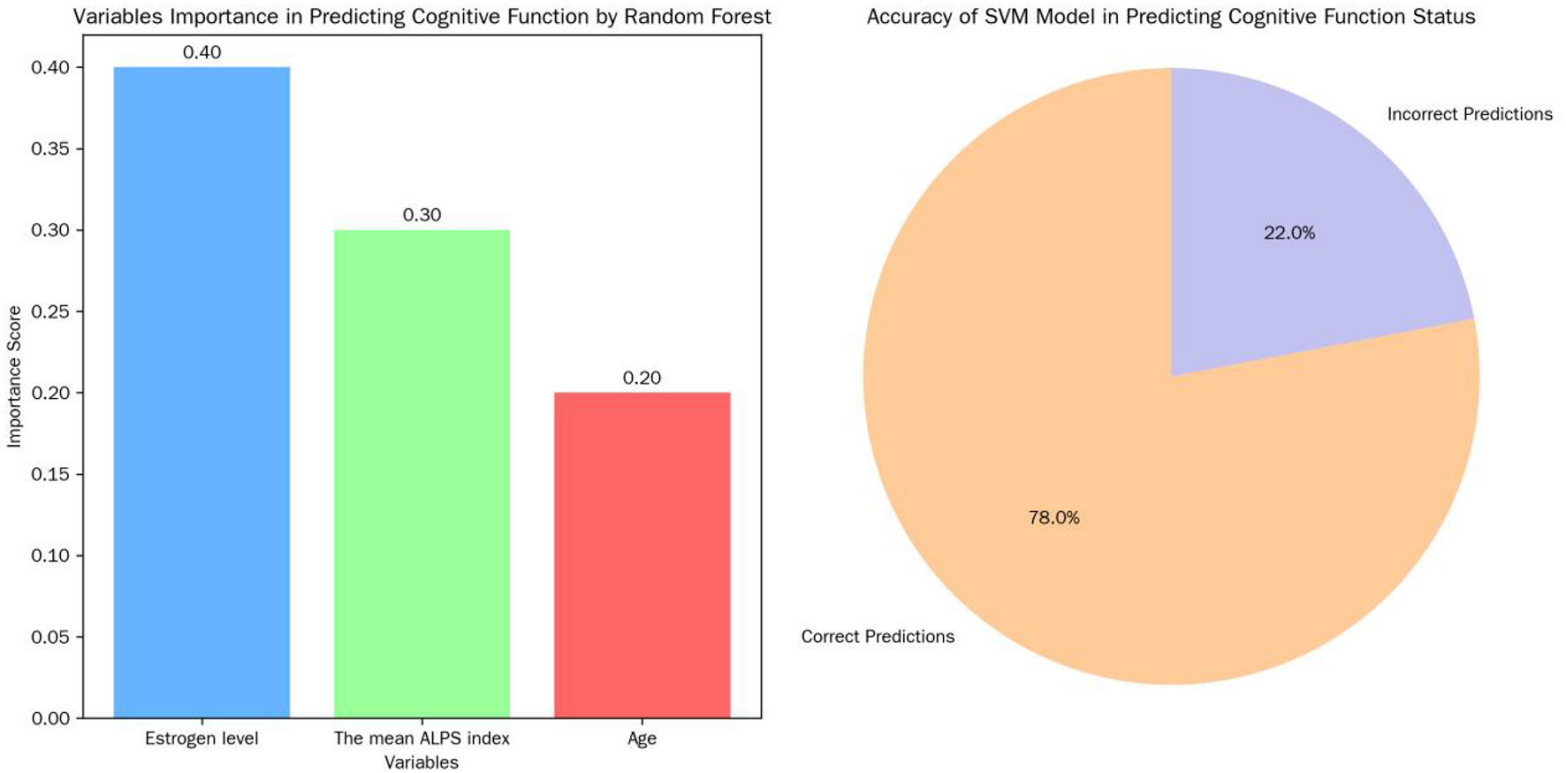
Variable Importance in predicting cognitive function and SVM model prediction accuracy.

Random forest algorithm ranked estrogen level, the mean ALPS index, and age as the top three most important variables in predicting cognitive function. The SVM model achieved an accuracy of 78% on the test set in predicting cognitive function status, indicating its potential in predicting cognitive function in postmenopausal women (Figure 8).

## 4 Discussion

### 4.1 Core mechanism: estrogen-driven lymphatic dysfunction mediates cognitive decline

Cognitive decline in postmenopausal women is strongly linked to estrogen deficiency (Nerattini et al., 2023), but the role of brain glymphatic dysfunction remains unclear. Our study demonstrates that the mean ALPS index—a biomarker of perivascular glymphatic function—fully mediates the association between estrogen levels and cognitive performance in executive tasks (e.g., Stroop, 2-back accuracy). This finding establishes a novel pathway: estrogen decline disrupts glymphatic clearance (as reflected by lower ALPS indices), leading to neurotoxic waste accumulation and cognitive impairment. This mechanism aligns with preclinical studies showing estrogen deficiency downregulates aquaporin-4, a key protein for perivascular fluid transport (Zhang et al., 2024; Kato et al., 2024), and clinical data linking ALPS indices to cognitive decline in aging (Kamagata et al., 2022; Taoka et al., 2017).

### 4.2 Hemispheric asymmetry and cognitive specialization

The selective decline in right hemisphere and global ALPS indices (but not left) mirrors known hemispheric specialization. The right hemisphere, involved in spatial and emotional processing, is more sensitive to estrogen fluctuations, while the left hemisphere (language/sequential tasks) shows relative resilience (Duboc et al., 2015; McIlvain et al., 2022; McEwen, 2002; McCarthy, 2008). This is supported by stronger correlations between right ALPS indices and cognitive accuracy (e.g., *r* = 0.588 for Stroop), which may reflect the right hemisphere's greater reliance on efficient waste clearance for complex cognitive demands (Huang et al., 2024). The non-significant left ALPS findings, though potentially limited by sample size (Cohen's *d* = 0.32), highlight the need for hemisphere-specific investigations in larger cohorts.

### 4.3 Multivariate and non-linear insights

MANOVA revealed that menopausal status independently influences ALPS indices, estrogen levels, and cognitive performance, even after controlling for age and education. The interaction between age and menopause on mean ALPS indices suggests that estrogen loss exacerbates age-related glymphatic decline, while premenopausal estrogen partially buffers this effect. This underscores the cumulative impact of hormonal and chronological aging on brain health (Marjoribanks et al., 2017; Guo et al., 2025).

Non-linear correlation analysis further revealed a threshold effect in ALPS-cognitive relationships: low ALPS levels showed strong associations with cognitive accuracy, possibly reflecting a critical waste clearance threshold for neural function. At medium/high ALPS levels, compensatory mechanisms (e.g., neural reserve) may blunt this relationship, highlighting the complexity of glymphatic-cognitive interactions (Kamagata et al., 2022; Huang et al., 2024; Lo and Rosenberg, 2009).

### 4.4 Advanced modeling and clinical implications

Structural equation modeling (SEM) confirmed the mediating role of ALPS indices and revealed an indirect negative effect of age on cognition via lymphatic dysfunction, reinforcing the centrality of glymphatic clearance in aging (Huang et al., 2025). Machine learning models (random forest/SVM) ranked estrogen, ALPS, and age as top predictors of cognitive decline, with SVM achieving 78% predictive accuracy. These tools may aid early risk identification, though mechanistic interpretation requires integration with traditional statistics (Richter-Laskowska et al., 2025).

Clinically, the ALPS index could serve as a non-invasive biomarker for monitoring postmenopausal cognitive health. Interventions targeting estrogen signaling (e.g., hormone therapy) or glymphatic function (e.g., aquaporin-4 agonists) may offer protective effects, particularly for women with low ALPS indices (Kato et al., 2024; Taoka et al., 2017; Huang et al., 2024).

### 4.5 Limitations and future directions

Despite these insights, the study has notable limitations. The cross-sectional design precludes causal inference, and the small sample size (*n* = 55, *n* = 15 premenopausal) may limit generalizability, though sensitivity analyses confirmed robust findings. Missing data on BMI, comorbidities, and medications introduce potential confounding, while educational disparities (postmenopausal: 10.63 ± 4.92 years vs. premenopausal: 16.07 ± 3.58 years) were statistically controlled but warrant longitudinal validation.

Future research should employ longitudinal designs with stratified sampling, incorporate genetic/lifestyle factors, and explore estrogen receptor-specific effects on glymphatic proteins. Larger cohorts will refine effect sizes and validate machine learning models, ensuring translation of ALPS-based biomarkers into clinical practice.

## 5 Conclusion

This study bridges estrogen physiology, brain glymphatic function, and cognitive health, identifying the ALPS index as a key mediator of postmenopausal cognitive decline. By integrating multivariate, non-linear, and machine learning approaches, we provide a comprehensive framework for understanding hormone-brain interactions. These findings not only advance mechanistic knowledge but also inform the development of early intervention strategies for a growing public health concern.

## Data Availability

The datasets presented in this article are not readily available because the datasets used and/or analyzed during the current study are available from the corresponding author upon reasonable request. Requests to access the datasets should be directed to Ningning Liu, ningnliunikita@163.com.
